# A core collection of pan-schizophrenia genes allows building cohort-specific signatures of affected brain

**DOI:** 10.1038/s41598-019-48605-3

**Published:** 2019-09-03

**Authors:** Qinglian Xie, WenWu Shen, Zhixiong Li, Ancha Baranova, Hongbao Cao, Zhe Li

**Affiliations:** 1 Out-patient Department and National Clinical Research Center for Geriatrics, West China Hospital of Sichuan University, Chengdu, Sichuan 610041 China; 2 The Third Department of Clinical Psychology, Karamay Municipal People’s Hospital, Karamay, Xinjiang 830054 China; 3 School of Systems Biology, George Mason University (GMU), Fairfax, VA 22030 USA; 4 Research Centre for Medical Genetics, Moscow, 115478 Russia; 5 Department of Psychiatry, First Hospital/First Clinical Medical College of Shanxi Medical University, Taiyuan, Shanxi 030001 China; 6 Department of Genomics Research, R&D Solutions, Elsevier Inc., Rockville, MD 20852 USA; 7 Mental Health Center and National Clinical Research Center for Geriatrics, West China Hospital of Sichuan University, Chengdu, Sichuan 610041 China

**Keywords:** Computational models, Gene expression

## Abstract

To investigate whether pan-schizophrenia genes could be leveraged for building cohort-specific signatures reflecting the functioning of the affected brain, we first collected 1,518 schizophrenia-related genes upon analysis of 12,316 independent peer-reviewed literature sources. More than half of these genes have been reported in at least 3 independent studies, and a majority (81.4%) were enriched within 156 functional pathways (p-values < 1e-15). Gene expression profiles of brain tissues were extracted from 14 publicly available independent datasets, and classified into “schizophrenia” and “normal” bins using dataset-specific subsets of core schizophrenia collection genes built with either a sparse representation-based variable selection (SRVS) approach or with analysis of variance (ANOVA)-based gene selection approach. Results showed that cohort-specific classifiers by both SRVS and ANOVA methods are capable of providing significantly higher accuracy in the diagnosis of schizophrenia than using the whole core genes (p < 3.38e-6), with relatively low sensitivity to the ethnic backgrounds or areas of brain biopsies. Our results suggest that the formation of consensus collection of pan-schizophrenia genes and its dissection into the functional components could be a feasible alternative to the expansion of sample size, which is needed for further in-depth studies of the pathophysiology of the human brain.

## Introduction

Schizophrenia is a highly heritable severe mental disorder characterized by abnormal behavior and a decreased ability to understand reality^1^. In the United States, the costs associated with schizophrenia impose a heavy financial burden on families and society^2^. Genetic factors, environmental factors, and life history play critical roles in the development of this mental condition^3–5^.

In recent years, many genetic markers/genes associated with schizophrenia have been uncovered; for many of these genes, their relation to schizophrenia was confirmed in at least two independent publications. Information concerning the genes somehow associated with schizophrenia may be used for modeling of this disease *in silico*, which, in turn, may facilitate the discovery of a minimally invasive biomarker for this disease, improve the diagnosis and contribute to the prevention of schizophrenia^6^. However, due to the heterogeneity of schizophrenia^7^ as well as varying penetrance of the genetic polymorphisms predisposing to schizophrenia in different populations^8,9^, the genes reported from one study usually lack replication in other studies, leading to a sizable pool of schizophrenia-associated genes after curation. Because of that, using an entire pool of schizophrenia-associated genes may not produce an adequate model to cover this disease in terms which are general enough to be applicable to all populations or to all variations in its symptoms. This conundrum may be solved either by producing multiple models of schizophrenia, each one fitting a particular need, or by building a “core” model of this condition, which may be later augmented with additional functional blocks, which may be either population- or symptom-specific.

To explore whether a “core” model of schizophrenia could be built, we conducted a comprehensive literature review to identify a curated pool of 1,518 schizophrenia-related genes. This work was undertaken under the assumption that only a small percentage of genes from an entire pool of schizophrenia-related genes are capable of differentiating any subset of schizophrenia patients as selected by particular symptom or other characteristics from a group of matched healthy controls. Then we employed a sparse representation-based variable selection (SRVS) algorithm for the further selection of the model components. In previous works, the SRVS algorithm has been demonstrated as an effective tool for the selection of genetic and imaging features under a condition when a considerable number of variables is studied in a relatively small number of samples^10^. Therefore, here, we employed the SRVS method to select cohort-specific genes from the pan-schizophrenia gene pool with the expectation to reach the best resultant differentiation of the schizophrenia patients from healthy controls within the cohort. To note, the purpose of this study is to test if the collected 1,518 pan-schizophrenia genes could be used as a gene pool to build core models for schizophrenia patients selected corresponding to different symptoms or other characteristics. Therefore, well-known or well-established validation methods should be employed rather than explore novel methods. Microarray gene expression data have been demonstrated effective for gene network-based classification^11^. Therefore, in this study, we used gene expression data unbiasedly-selected from a publicly available database (GEO: Gene Expression Omnibus) for the gene selection and validation approaches. However, instead of analyzing the whole genome, the expression data-analysis will be based on the 1,518 pan-schizophrenia gene pool, which will reduce noise and increase diagnosis efficiency and accuracy. The hypothesis is that, although the use of all genes described in the literature will not give good classifiers, the pan-schizophrenia pool curated from previous large-scale studies contains majority schizophrenia-related genes.

This approach may lead to highlighting cohort-specific gene markers identification targeting accurate diagnosis that is necessary for precision treatment. The formation of consensus collection of pan-schizophrenia genes and its dissection into the functional components provide a feasible alternative to expansion of sample size. We summarize the novelty of this study as follows. (1) As far as we know, this is the first study curating a 1,518-pan-schizophrenia gene pool upon large-scale literature-based analysis of 12,316 schizophrenia references. (2) We proposed an effective and efficient approach (pan-schizophrenia gene pool-based gene expression data analysis) to identify cohort-specific gene markers targeting accurate diagnosis that is necessary for precision treatment. (3) We proposed a potentially feasible alternative to expansion of sample size in the identification of effective gene markers needed for precision treatment.

## Results

### Analysis of knowledge-based connections between each of schizophrenia-associated genes and schizophrenia

In the course of comprehensive literature data mining effort, we collated a total of 12,316 scientific articles reporting 1,518 genes associated with schizophrenia. The full list of these genes, and the supporting references for each gene-disease relationship, including title, publication year, authors, their affiliations, and relevant sentences from the full-text manuscript may be found in the in the table **SCZ_2018**→SCZ_Genes, which is online available at http://gousinfo.com/database/Data_Genetic/SCZ_2018.xlsx.

**Figure 1:**
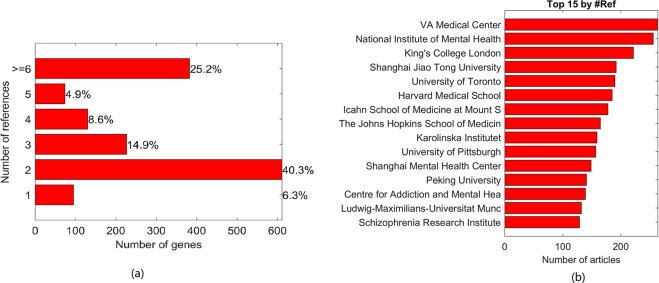
Knowledge-based connections between each of schizophrenia-associated genes and schizophrenia. (**a**) Bar graph which reflects relative shares of schizophrenia-associated genes supported by varying amount of references. (**b**) Top 15 research institutions which contributed to uncovering the relationships between each gene and schizophrenia.

Figure 1(a) presents the percentages of genes supported by various amounts of publications. Over half of the 1,518 genes have been reported in at least 3 independent studies, making them less likely to turn false positives. Figure 1(b) presents the Top 15 affiliations of the research teams endorsing the data. These 15 affiliations account for only 0.4% (15/4172) of a total amount of research institutions contributed to the identification of schizophrenia-associated genes while covering near half (46.8%) of the entire gene set. For the detailed analysis of these references, please refer to **SCZ_2018**→Ref4SCZGenes.

## GSEA Results

A complete list of significantly enriched pathways/gene sets (N = 156, p-value < 6.4e-16) could be found in **SCZ_2018→GSEA**. A total of 1,236 out of the 1,518 schizophrenia-related genes (81.4%) were found to participate in one or another of significantly enriched pathways. In Table 1, we present the Top 10 pathways/groups, each with a p-value of less than 1.3e-103. Of note, the top 10 pathways encompassed a total of 635 out of 1,518 schizophrenia-associated genes (41.83%). A majority of significantly enriched pathways have been implicated in the pathogenesis of schizophrenia in previous studies, thus supporting that the relationships between the genes and the disorder were mined correctly. The pathway analysis was conducted using Pathway Studio against the Gene Ontology (GO).

**Table 1:** Top 10 pathways/functional groups with an overrepresentation of genes previously described as associated with schizophrenia.

Name of the process or pathway	GO ID	# of Entities	Overlap	p-value
behavior	0007610	713	277	7.2e-140
synapse part	0044456	715	268	3.2e-135
single-organism behavior	0044708	513	218	6.0e-118
cell communication	0007154	932	290	7.8e-118
neurotransmitter receptor	0030594	230	93	1.1e-116
modulation of synaptic transmission	0050804	364	184	1.7e-115
cell body	0044297	630	226	2.9e-108
synaptic signaling	0099536	356	172	1.3e-103
chemical synaptic transmission	0007268	356	172	1.3e-103
anterograde trans-synaptic signaling	0098916	356	172	1.3e-103

These Top 10 pathways/functional groups cover a total of 636 genes with enrichment p-values < 1.3e-103.

## Classification Results

The information of the 14 selected datasets was provided in Table 2, as bellow.

**Table 2:** Key descriptors of 14 schizophrenia-related datasets selected for this study.

	N of Cases/Controls	N of genes in common with the curated dataset of schizophrenia-related genes	Specimen studied	Population
GSE12649	35/34	1276	prefrontal cortex	Japan
GSE12654	13/15	1112	prefrontal cortex	Japan
GSE12679	16/11	1440	dorsolateral prefrontal cortex	United Kingdom
GSE17612	28/23	1440	BA10	United Kingdom
GSE21138	30/29	802	prefrontal cortex	USA
GSE21935	23/19	1440	BA22	United Kingdom
GSE26927	10/55	1392	Multiple Brodmann areas	United Kingdom
GSE35974	44/50	1469	parietal cortex	China
GSE35977	51/50	1469	parietal cortex	China
GSE35978	95/100	1469	parietal cortex	China
GSE53987	48/55	1440	prefrontal cortex (BA46)	USA
GSE62191	29/30	693	frontal cortex	Brazil
GSE87610	65/72	1406	prefrontal cortex	USA
GSE93987	67/106	1429	prefrontal cortex	USA

Table 3 summarizes the results of leave-one-out (LOO) cross-validation of the two applied techniques of gene ranking, SRVS and ANOVA, in each of 14 datasets, including the maximum CR, amounts of genes in the top classifier, and permutation p-values. For each given dataset, the constituents of optimal classifiers selected by SRVS and ANOVA differed substantially. The classifiers selected by the same algorithm being applied to different dataset also differ, reflecting both underlying differences in gene expression profiles between various brain tissues and between populations of patients. Table 3 shows that both SRVS and ANOVA based classifier led to significant classification ratio (CR) compared to non-core based classifier (the averaged permutation p-value < 6.30e-3 and < 5.00e-4 for SRVS and ANOVA, respectively). Across all 14 datasets, using the entire core collection of schizophrenia genes over a randomly selected set of genes with a similar size presented negligible advantage (p-value = 0.41 ± 0.37). For each of the 14 expression datasets, the constituents of optimal classifiers (a list of gene symbols) are presented in SCZ_2018→Classifiers_SRVS and SCZ_2018→Classifiers_PValue, corresponding to SRVS and ANOVA selected classifiers (gene markers), respectively.

**Table 3:** SRVS and ANOVA analysis of optimal gene expression classifiers in 14 schizophrenia-related datasets.

	CR1	CR2	CR3	G1	G2	G3	P1	P2	P3
GSE12649	89.86	75.36	59.42	141	1	1276	<2.00e-4	<2.00e-4	0.12
GSE12654	92.86	89.29	60.71	163	10	1112	<2.00e-4	<2.00e-4	0.45
GSE12679	85.19	100	66.67	30	52	1440	<2.00e-4	<2.00e-4	0.07
GSE17612	90.20	88.24	52.94	30	60	1440	<2.00e-4	<2.00e-4	0.17
GSE21138	86.44	74.58	66.10	31	19	802	<2.00e-4	2.80e-3	0.64
GSE21935	88.10	85.71	64.29	675	27	1440	<2.00e-4	<2.00e-4	2.52e-2
GSE26927	93.85	89.23	69.23	64	40	1392	<2.00e-4	1.60e-3	0.93
GSE35974	89.36	84.04	64.89	28	14	1469	<2.00e-4	<2.00e-4	0.92
GSE35977	82.18	77.23	71.29	290	107	1469	<2.00e-4	2.00e-4	5.00e-3
GSE35978	53.38	71.28	52.31	27	17	1469	8.06e-2	<2.00e-4	0.24
GSE53987	55.34	68.93	52.43	58	2	1440	<4.80e-3	4.00e-4	1.00
GSE62191	79.66	76.27	54.24	44	11	693	<2.00e-4	<2.00e-4	0.48
GSE87610	90.51	86.13	74.45	517	238	1406	<2.00e-4	<2.00e-4	0.02
GSE93987	87.28	86.71	75.72	108	9	1429	<2.00e-4	<2.00e-4	0.65

**Note:** CR: classification accuracy. CR1: CR by SRVS Score; CR2: CR by PValueSCore (ANOVA); CR3: CR built upon all the 1518 schizophrenia-related genes that were also included in a dataset; G1: Amount of genes selected by SRVS Score; G2: Amount of genes selected by PValueSCore (ANOVA); G3: Amount of all schizophrenia-related genes in each dataset; P1: Permutation p-value by SRVS Score classifier; P2: Permutation p-value by PValueSCore (ANOVA) classifier; P3: Permutation p-value after using all schizophrenia-related genes present in a given dataset as a classifier.

**Figure 2:**
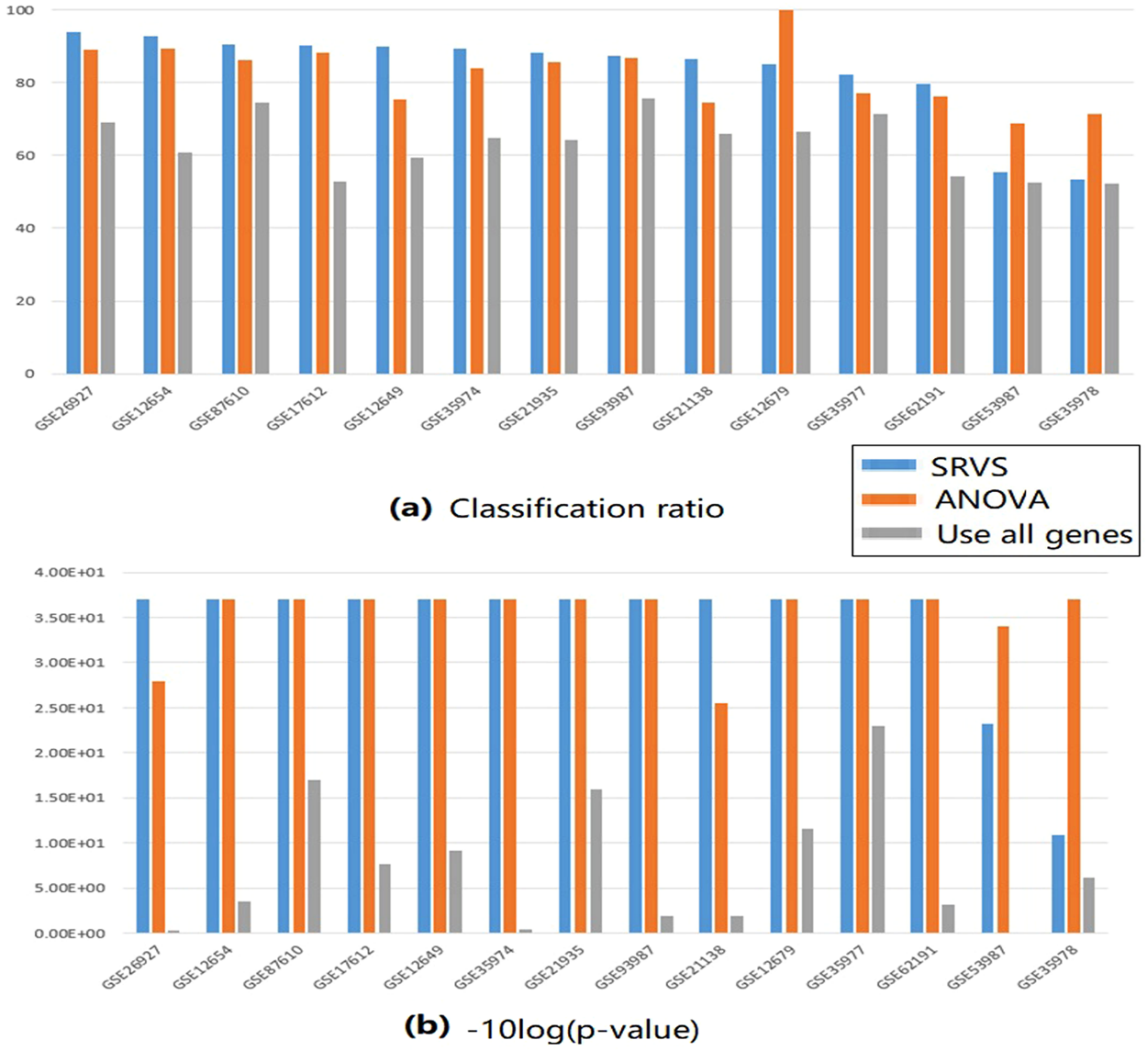
Bar graphs presenting the performance of SRVS, ANOVA and pan-signature classifiers in 14 expression datasets. (**a**) Classification ratio; (**b**) Permutation p-value (−10 * log (p-value)).

**Figure 3:**
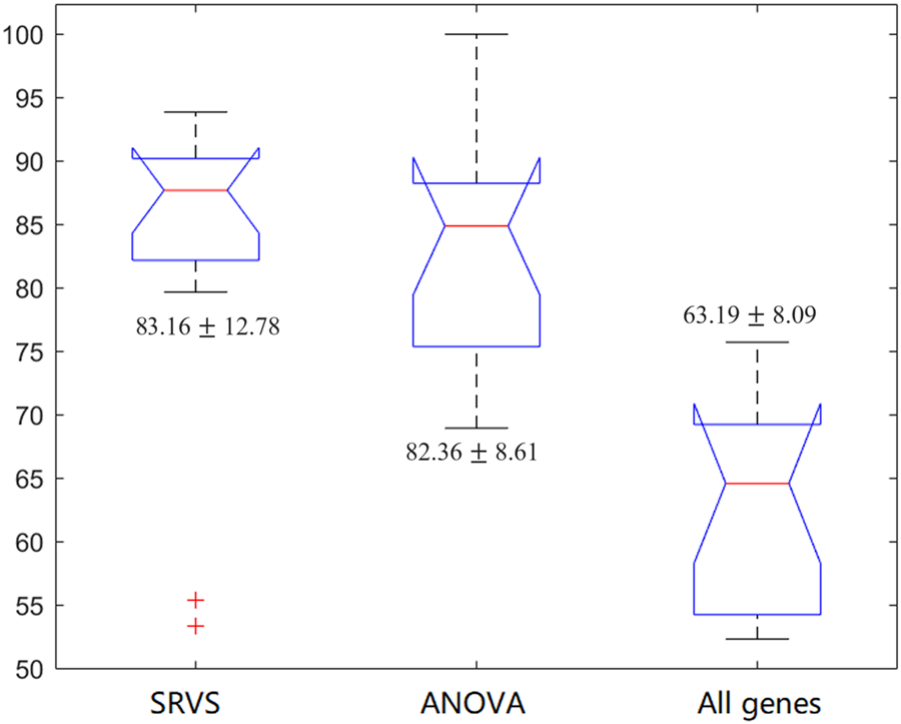
Box-plot of the performance of classifiers built by SRVS and ANOVA ranking procedures as well as by pan-signature classifiers across 14 datasets.

Figure 2 presents a bar graph which reflects dataset-specific CRs and p-values for classifiers obtained by SRVS-based and ANOVA-based selection, as well as by utilizing all available schizophrenia-associated genes detected within each dataset. Both SRVS and ANOVA classifiers significantly outperform classifier built upon entire schizophrenia signature (p < 3.38e-6; CR = 83.16 ± 12.78, 82.36 ± 8.61 and 63.19 ± 8.09 for SRVS classifier, ANOVA classifier, and entire signature, respectively (Fig. 3). Moreover, classifiers built upon entire schizophrenia-related signature (N = 1,518 genes) have not presented an advantage over the pan-signature classifiers built upon an equivalent amount of randomly selected genes (CR = 0.41 ± 0.37). No significant differences between SRVS and ANOVA based CRs were detected (p-value = 0.85) (Table 3).

### Comparison of the gene sets selected as best classifiers by SRVS and ANOVA procedures

**Figure 4:**
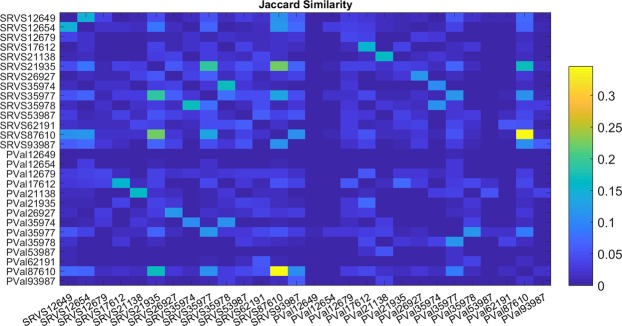
Jaccard Similarity of the dataset-specific classifier gene sets selected by SRVS and ANOVA techniques. SRVS outputs are labeled as ‘SRVS with respective GSE ID’; ANOVA outputs are labeled as ‘PVal with respective GSE ID’. Diagonal entries represent the Jaccard Similarity of a study with itself, which is always equal to one. The ones on the diagonal line were set to zeros.

The gene sets selected as classifiers in 14 different studies with SRVS and ANOVA were compared using Jacquard Similarity score (JSScore)^12^, as shown in Fig. 4. The genes included (the classifiers) in each gene set and respective JSScores are provided in **SCZ_2018** (Classifiers_SRVS, Classifiers_PValue, and JaccardSimilarity, respectively).

As shown in Fig. 3, dataset-specific classifier gene sets hardly overlap (JSScore < 0.35). This unexpected finding may be explained by the factors playing a role only in a specific study, for example, the type of the brain tissue profiled for its mRNA profile. To test if small sizes of detected gene set overlaps were caused by any of the factors intrinsic to each of the studied cohorts, a 3-way ANOVA analysis was conducted. Table 4 shows that none of these three evaluated parameters exerted a significant influence on the JSScore (p-value > 0.40).

**Table 4:** The output of 3-way ANOVA test for the influence of cohort-specific factors on Jacquard similarity of dataset-specific classifier gene sets.

Source	Sum Sq.	d.f.	Mean Sq.	F	P > F
Brain Region	0.0098	5	0.0020	0.060	1.00
Ethnicity	0.082	13	0.0063	0.18	1.00
SRVS or ANOVA	0.024	1	0.024	0.70	0.40
Error	26.70	764	0.035		
Total	26.82	783			

## Discussion

Schizophrenia affects approximately 1 in 100 people worldwide^1^. During the past decade, many studies have aimed to extract genetic contributors to schizophrenia phenotypes. A typical output of any such research presented a differentially expressed gene set substantially differing from that obtained by analyzing any other independent cohort, a feature commonly explained by cohort-to-cohort differences in terms of its size, ethnicity, the characteristics of the disease itself, and the technical differences in the data processing. We hypothesize that these minimally-overlapping gene sets, however, maintain a strong functional association with schizophrenia as well as with each other, and provide the pathophysiological underpinning of the disease en masse.

In this work, all schizophrenia signature genes collected across more than 12,000 automatically parsed research manuscripts were combined into one database. Initial gene set enrichment analysis (GSEA) of the entire collection of schizophrenia-related genes sorted them into 156 molecular pathways/functional groups. Among them, 18 pathways/gene sets encompassing a total of 644 unique genes were related to the nervous system, 3 pathways/gene sets with 163 unique genes were related to brain function development, and 2 pathways/gene sets with 277 unique genes were related to behavior. These connections were expected. For instance, out of 713 genes comprising GO: behavior (GO ID: 0007610), a total of 277 genes were related to schizophrenia; many of these genes also harbor variant sequence positions independently associated both with the behavioral response to stress and to psychotic symptoms^13^. There was also a 256-gene intersection between pan-schizophrenia signature and 715 genes comprising GO: synaptic part (GO ID: 0044456); respectively, deregulation of synaptic activity is one of most recognized contributors to schizophrenia etiology^14,15^.

It is, however, expected that only a subset of pan-schizophrenia gene collection will be associated with particular features of the disease, or be expressed in the particular region of the brain, or be involved in the development of this disease in individuals with particular ethnic background. To test this hypothesis, further evaluation of pan-schizophrenia gene collection was performed through independent rounds of case/control classifications conducted with two different algorithms for gene selection, namely, SRVS and ANOVA, in fourteen publicly available gene expression datasets. These algorithms aid in ranking potential classifiers by arranging them in a list according to SRVSScore or PValueScore, respectively. Both algorithms also provide for an optimal amount of classifiers which ensures the best accuracy of resultant classification.

Notable, amounts of genes selected by either SRVS or ANOVA methods showed significant variation across datasets (Table 3), pointing at other factors that affect the composition of an optimal set of features at play. As shown in Table 2, the fourteen datasets were collected by profiling patient populations with different ethnic backgrounds, moreover, the areas of brain biopsies were different as well. Nevertheless, these very obvious variables were found to have a negligent effect on the variation in the composition of best-classifying signatures across datasets (Table 3).

It seems that low robustness of classifier signature in schizophrenia is inherent to the nature of analyzed data, as it relates to the small number of samples comprising each discovery dataset. This problem has been extensively studied in relation to predictive signatures of cancer progression^16,17^. In particular, several published datasets on breast carcinoma were re-analyzed to show that achieving the desired overlap of 50% between two predictive gene signatures, and at least several thousand patients should be enrolled in each discovery cohort^18^. Understanding of sample set limitations gained in course of these studies was later translated into a number of national and international biobanking initiatives resulting in accrual of a substantially larger cohort of patients for a majority of common cancers^19^. It is, however, not expected that the collection of post-mortem needle biopsy or whole-brain specimens well achieve requisite numbers in either short-term or mid-term perspective. Therefore, an analysis of available or yet-be-available schizophrenia-related datasets is expected to produce non-robust signatures with characteristics similar to that described for early discovery datasets mined for outcome-associated signatures. In particular, according to analysis published in^17^, each of these datasets would be expected to include (1) many hundreds of genes correlated with the presence of schizophrenia; (2) many hundreds of genes with approximately same degree of correlation to presence of schizophrenia or any of its isolated features; (3) these correlations would be expected to vary dramatically when measured over different subsets of patients within the same dataset.

In light of this prediction, the formation of consensus collection of pan-schizophrenia genes and its dissection into the functional components provide a feasible alternative to expansion of sample size. Our study suggested that cohort-specific classifiers selected from the pan-schizophrenia gene collection are capable of providing high accuracy in the diagnosis of schizophrenia according to expression signature in the brain, with relatively low sensitivity to a region of sampling. With that, our study provides for an interesting avenue for further in-depth studies of the pathophysiology of the human brain.

Our results guaranteed several further studies. First, we employed 14 datasets from GEO. Observations from this study need to be validated using more datasets of different data type (e.g., GWAS data) from other database repositories (e.g, ArrayExpress: https://www.ebi.ac.uk/arrayexpress). In addition, we used two methods for gene selection (SRVS and ANOVA). Other feature selection methods can be employed when replicating the workflow.

## Materials and Methods

**Figure 5:**
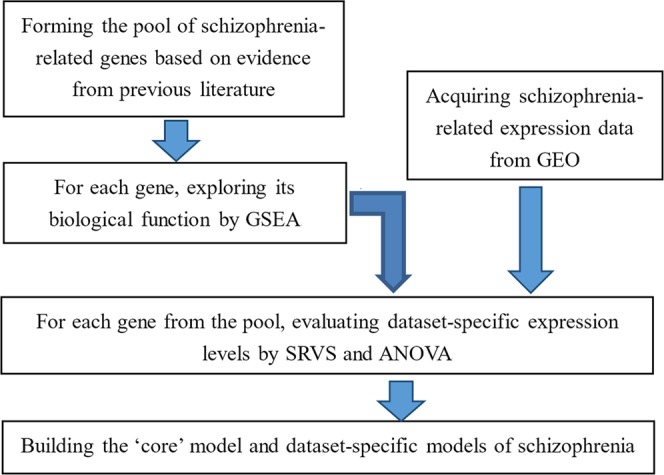
Diagram of the workflow for building a “core” model of schizophrenia.

To identify all possible schizophrenia-related genes, a large-scale systematic gene-disease relation data analysis was conducted in Pathway Studio environment (www.pathwaystudio.com)^20^. For each of these genes, its expression levels were investigated in 14 independent schizophrenia-related datasets that are publically available from gene expression omnibus (GEO; www.ncbi.nlm.nih.gov/geo/), following the workflow as follows: (1) quantitative evaluations were obtained using two statistical methods: sparse representation based variable section (SRVS)^10^ and one-way analysis of variant (ANOVA); (2) to select the best dataset-specific subset of genetic contributors, a case/control classifications, followed by a leave-one-out (LOO) cross-validations, have been performed. The diagram of the workflow of this study is presented in Fig. 5.

### Relationships between each gene and the schizophrenia

The literature-based search for schizophrenia-related genes was performed in Pathway Studio environment (www.pathwaystudio.com). Each of the uncovered relationships was supported by one or more supporting references. Genes were ranked by the total number of references linking them to schizophrenia. The list of these genes and the relationships between these genes and schizophrenia has been listed in the Table **SCZ_2018** (http://gousinfo.com/database/Data_Genetic/SCZ_2018.xlsx).

### GSEA analysis of schizophrenia-related genes

To explore the functionality of the literature-mined schizophrenia genes and identify their related pathways, a Gene Set Enrichment Analysis (GSEA) has been conducted using Pathway Studio, with an input of the complete list of identified genes (N = 1,518), and the outputs of the enriched pathways/gene sets and the related statistics. The GSEA has been conducted against several pathway databases, including Gene Ontology (GO), Pathway Studio Ontology and Pathway Studio Pathways.

### Selection of expression datasets

In this study, we used 14 schizophrenia-related expression datasets presented in Table 2. The datasets were selected by using the Illumina Correlation Engine (http://www.illumina.com) with keyword ‘schizophrenia’. All datasets are publicly available at GEO. The data selection criteria were as follows: (1) The organism is *Homo sapiens*; (2) The data type is RNA expression by array; (3) The sample size is no less than 30 specimens; (4) The study has case-control design; (5) The dataset and its format files are publically available; (6) Specimens represent various regions of the brain. From each dataset, expression data for the normal controls and for schizophrenia patients were extracted and then used for case/control classification. Before classification, the expressed gene sets were trimmed to include only the genes presented in the curated dataset of schizophrenia-related genes SCZ_2018→SCZ_Genes. To note, the gene identification process was based on 12,316 scientific articles, which is independent of the 14 datasets selected.

### Gene marker selection

A sparse representation-based variable selection (SRVS) algorithm has been described in details previously^10^. In each gene expression dataset, all detected mRNAs also present in manually curated schizophrenia gene database were ranked by SRVS algorithm. For each gene, a sparse weight, named “SRVS Score”, was assigned by SRVS. The gene vector, composed of the top *n* genes selected by SRVS, has been utilized as the dataset-specific classifier for cases and controls, where *n* is the number of genes corresponding to the maximum classification ratio (CR) as defined in Eq. (1).

1 $$classification\,Ratio\,(CR)=\frac{\#correctly\,classification\,subjects}{\#total\,subjects}$$

The classification approach is described as follows. For a given data set, the schizophrenia-associated genes were ranked in descending order, based on their SRVS Scores. Subsequently, a Euclidean distance-based multivariate classification^10^ was performed for each dataset, followed by a leave-one-out (LOO) cross-validation procedure^21^. For each run of LOO, the gene expression level of one sample within a dataset was used for testing, while the expression data of the rest samples were used as a training set. The inputs of the classifier were the expression values of the top *n* (*n* = 1, 2 …) genes; in this way, the CRs of the top *n* genes were determined. A permutation of 5,000 runs was then conducted to test the hypothesis that randomly selected gene sets of the same size can reach equal or higher CR, and the permutation P-values (number of runs with equal or better CRs over the number of total runs) were calculated. The gene vector that generated the highest CR was considered the best dataset-specific classifier, and, therefore, selected.

Following the same process, the best gene vector selected by the traditional ANOVA approach was identified for each dataset. For comparison purposes, CR baselines were generated using randomly selected gene sets of n (*n* = 1, 2 …) genes. For each point of the CR baseline, the value was the mean of 300 CRs, which were produced by randomly selected dataset-specific sets of any genes detected as expressed within this dataset.

## Data Availability

The processed data required to reproduce these findings are available upon request of the corresponding author.
